# Restoration Contexts and Trends in the Brazilian Atlantic Forest: Evidence from Science and Practice

**DOI:** 10.1007/s00267-026-02528-8

**Published:** 2026-06-12

**Authors:** Sofia Corradi Oliveira, Britaldo Silveira Soares-Filho, Getúlio Fonseca Domingues, Ubirajara Oliveira

**Affiliations:** 1 Graduate Program in Analysis and Modeling of Environmental Systems, Institute of Geosciences, Federal University of Minas Gerais, Belo Horizonte, Minas Gerais Brazil; 2 Centre for Remote Sensing, Institute of Geosciences, Federal University of Minas Gerais, Belo Horizonte, Minas Gerais Brazil

**Keywords:** restoration strategies, land-use planning, environmental and social benefits, policy and governance, systematic review

## Abstract

Large-scale restoration in the Brazilian Atlantic Forest is expected to recover biodiversity, carbon stocks, and other ecosystem functions while also supporting rural livelihoods. Yet the evidence supporting these claimed outcomes remains uneven, especially when distinguishing reported outcomes from causal evidence based on baselines, controls, counterfactuals, or long-term monitoring. We assessed peer-reviewed studies and a contextual set of documented initiatives to synthesize reported environmental, social, and economic findings together with governance and financing conditions associated with restoration in the biome. We also distinguished between direct field measurements, model-based estimates, descriptive reporting, and perception-based evidence. Across the reviewed sample, direct evidence was strongest for carbon and biodiversity, weaker for soil, and much more limited for hydrological and social effects. On-site studies often reported partial recovery relative to mature forests, especially in naturally regenerated areas, while model-based studies were more common in identifying where restoration might deliver multiple gains or lower opportunity costs. Social and financial evidence focused mainly on jobs, income, participation, opportunity costs, and implementation costs, but remained heterogeneous and rarely relied on standardized indicators. Governance was reported mainly through implementation conditions, including technical support, incentives, local participation, and coordination across actors and scales. The reviewed literature now covers more topics, but evidence remains uneven across ecological, social, financial, and governance dimensions. These gaps still limit comparison across cases and complicate restoration planning, monitoring, and policy alignment in the Atlantic Forest.

## Introduction

Land degradation and habitat fragmentation, mainly driven by deforestation, agricultural expansion, and other forms of land-use change, are reducing biodiversity, ecosystem functions, and soil productivity worldwide. Climate change adds further pressure through altered rainfall regimes, higher temperatures, and more frequent extreme events (Haddad et al. 2015; UNCCD 2022a, b). In this context, ecosystem restoration has become part of major international agendas, including the UN Decade on Ecosystem Restoration (2021–2030), the Kunming-Montreal Global Biodiversity Framework, and the Global Stocktake of the Paris Agreement (UNEP and FAO 2020; CBD 2022; UNFCCC 2023).

Brazil has translated this broader agenda into restoration commitments, including 12 million hectares by 2030, and is increasingly focusing on spatial planning and policy innovation (Brazil 2024). The Brazilian Atlantic Forest is central to this agenda. Although the biome retains only a fraction of its original native vegetation, it remains a global biodiversity hotspot and a major focus of restoration practice and planning, with expected environmental and socio-economic gains across highly modified landscapes (Brancalion et al. 2019b). Recent assessments also point to a growing restoration and bioeconomy agenda in Brazil, linked to emerging investment and financing mechanisms, job creation, and new opportunities to generate value from forest products and ecosystem services. The agenda still faces major constraints related to value-chain development, market access, public policy support, and the need to reconcile economic viability with biodiversity and social goals (Afonso 2022; Krainovic et al. 2025; Piotrowski et al. 2025). An additional challenge is that benefits are not only unevenly measured but also unequally distributed. Who benefits from restoration, and under what governance and financing arrangements, can influence local support, participation, and the long-term durability of restoration efforts (Krainovic et al. 2025; Piotrowski et al. 2025).

Yet the evidence used to guide restoration remains fragmented. Outcomes, defined as reported effects associated with restoration, are not always directly measured, and their monitoring remains inconsistent. Governance and financing, treated separately as implementation conditions, are also often described without a common structure. These inconsistencies limit comparison across cases and make it harder to identify which claims are supported by measured outcomes, modeled estimates, or implementation records. More broadly, mismatches between science, policy, and practice remain common, and knowledge gaps persist for results that vary over time and across landscapes, especially biodiversity and water regulation (Dib et al. 2023; Evans et al. 2023; Fischer et al. 2023; Hua et al. 2024). These difficulties are compounded by land tenure insecurity, poverty, and weak governance structures (IPCC 2019; Loveridge et al. 2022; Romanelli et al. 2024). This calls for evaluation frameworks that integrate ecological, social, and institutional dimensions (Borda-Niño et al. 2020; Simonson et al. 2021).

Restoration science has also changed. Earlier views often centered on recovering historical vegetation and stable ecological trajectories (Hobbs and Harris 2001; Suding 2011). More recent work gives greater weight to adaptive management, dynamic baselines, and the realities of working in transformed landscapes (Gann et al. 2019). Restoration is now more often treated as a continuum of strategies, from passive regeneration to active planting, shaped by landscape context, social goals, and governance arrangements (Chazdon et al. 2015; Reid et al. 2018; Brancalion and Holl 2020). The evidence base now includes implementation choices, trade-offs, and context-dependent conditions alongside biophysical responses.

Previous reviews have examined specific dimensions of restoration in the Atlantic Forest and in Brazil, including bibliometric trends, knowledge gaps, historical trajectories of restoration science and practice, biodiversity responses, economic costs and benefits, multifunctional restoration, and monitoring indicators (Oliveira et al. 2021; Zupo et al. 2022; Romanelli et al. 2022, 2024; Rother et al. 2023; Santos et al. 2023b; Schimetka et al. 2024). These studies show that restoration research in the biome has expanded, but they mostly focus on specific evidence domains or on the field’s development. Few syntheses examine the environmental, social, financial/economic, and governance dimensions together and specify whether claims are based on field measurements, model estimates, perception-based data, descriptive reporting, or documented initiatives. This distinction helps clarify which restoration outcomes are directly measured, which are estimated, and which are mainly documented as implementation claims.

In this study, we review restoration in the Brazilian Atlantic Forest to synthesize reported environmental, social, and economic findings together with governance and financing conditions described in peer-reviewed studies and documented initiatives. We also characterize how this evidence base is distributed across publication patterns, thematic concentrations, study areas, and evidence types. By distinguishing measured outcomes, modeled estimates, perception-based evidence, descriptive reporting, and implementation conditions, we aim to clarify what the current evidence base can support, where it remains fragmented, and what this means for restoration evaluation, planning, and policy alignment. We use this distinction to separate evidence of implementation and reported outcomes from stronger causal evidence of restoration effects.

## Methods

### Review Scope and Definitions

We focus on the Brazilian Atlantic Forest biome, which covers approximately 110 million ha (IBGE 2019) and includes diverse native vegetation types (IBGE 2024) across varied socioeconomic contexts. The biome has become a major focus of restoration practice and research due to its concentration of endangered species, vulnerability to climate change, and role in regional water regulation (Pinto et al. 2014; Molin et al. 2017; Ribeiro et al. 2020; Zanini et al. 2021; Anunciação et al. 2023; Lima et al. 2024; Costa et al. 2024).

At the regional level, restoration efforts across the Atlantic Forest of Argentina, Brazil, and Paraguay were recognized by the United Nations as one of the inaugural World Restoration Flagships, through the trinational restoration effort coordinated by the Pact for the Restoration of the Atlantic Forest (UNEP 2022). The biome nevertheless continues to face forest loss and degradation, reinforcing the need to track native vegetation and restoration trajectories over time (Amaral et al. 2025).

In this review, we use restoration as shorthand for ecological restoration actions and recovery processes aimed at assisting the reestablishment of native vegetation in the Brazilian Atlantic Forest biome and adjacent ecotonal areas, as described in the literature as transition zones (Gann et al. 2019). We recognize that ecosystem restoration is broader, including rehabilitation, forest landscape restoration, land restoration, and actions to reduce ongoing degradation (UNEP and FAO 2020). The scope includes forest formations and other native vegetation types represented within the biome.

For analytical purposes, interventions were defined as discrete implemented or modeled restoration actions intended to promote native vegetation recovery, including site-level actions, projects, programs, and management measures in which a restoration action was specified. Initiatives were defined as broader institutional, collaborative, or policy arrangements that support, organize, or coordinate restoration across actors, sites, or scales.

### Methodological Approach

**Fig. 1:**
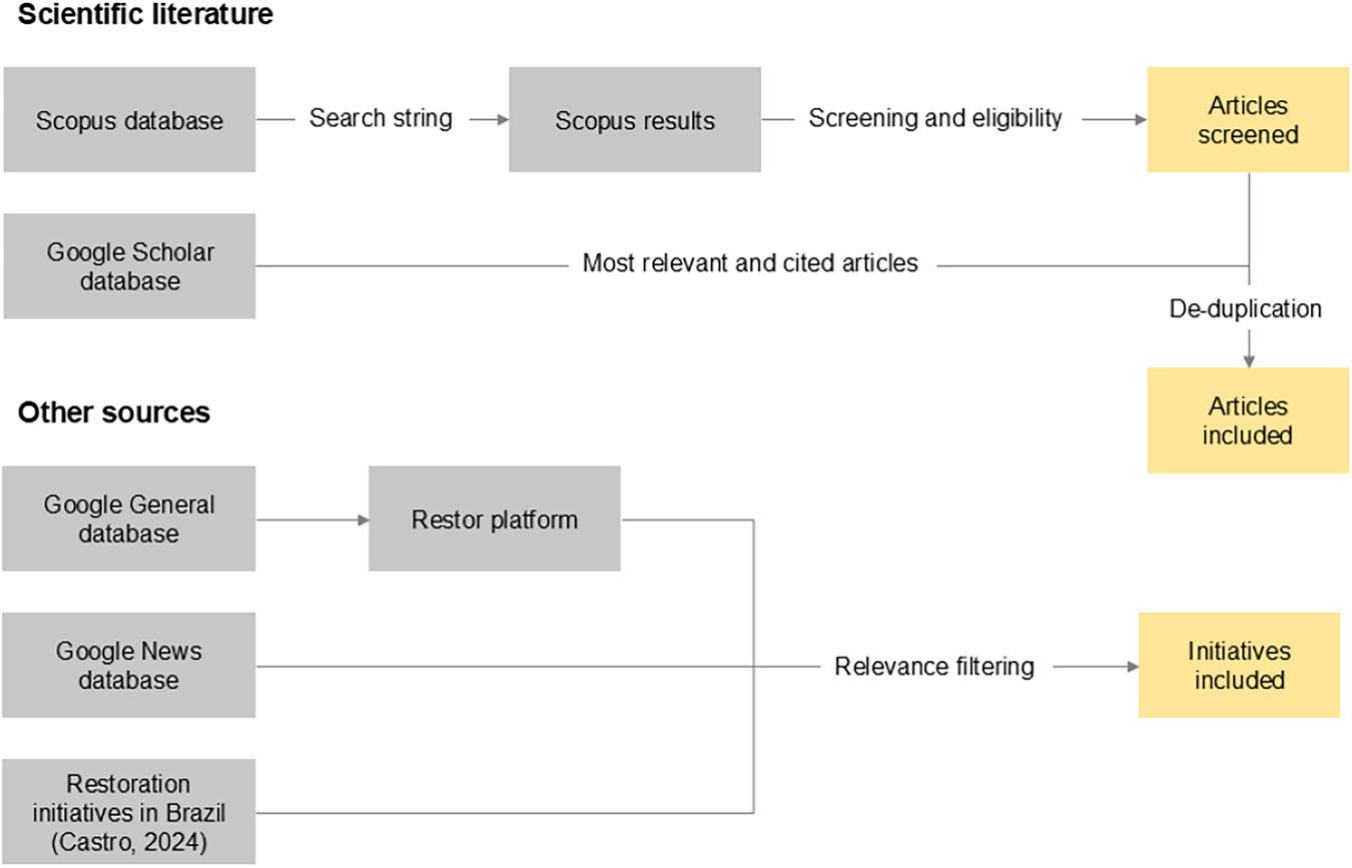
Workflow for identifying, screening, and including peer-reviewed studies and documented sources on restoration initiatives

Our methodological approach, outlined in Fig. 1, followed evidence synthesis guidelines (CEE 2022) centered on a Scopus search conducted in August 2024. We built the search string using the PICOC framework (Population, Intervention, Comparison, Outcome, and Context) (Hosseini et al. 2024) and refined it after testing for scope and accuracy. Table 1 presents the categories and keywords used, combined with the Boolean operator AND. We included articles, conference papers, and book chapters written in English, Portuguese, and Spanish.

**Table 1:** Keyword combinations used in the search

Category	Keywords and operators
Activity	restor* OR reforest* OR regenerat* OR revegetat* OR recovery
Country	brazil* OR brasil*
Region	“atlantic forest” OR “atlantic rainforest” OR “atlantic rain forest” OR “mata atlântica”
Environmental aspects	“ecosystem service*“ OR “environmental service*“ OR carbon OR biodiversity OR water OR soil
Financial and socioeconomic aspects	forestry OR silviculture OR agroforest* OR “agro-forest*“ OR payment OR credit* OR benefit* OR income OR economic* OR soci*

We defined the eligibility criteria as requiring studies to (1) be located in Brazil’s Atlantic Forest or in adjacent transition zones explicitly described as such in the source, and (2) report at least one restoration-related finding or implementation condition within an environmental, social, financial/economic, or governance dimension. These findings could be presented either as measurable variables, that is, quantified metrics or estimates, or as categorized information, that is, clearly described classes, types, or qualitative groupings that could be consistently recorded during data extraction. After screening and full-text review, we extracted and indexed data on intervention type, geographic scope, time frame, replicability, and the analytical dimension under which each finding or condition was classified. A comparable breakdown of intervention type, area, and land tenure was possible only for a subset of documented initiatives, as these descriptors were inconsistently reported across the peer-reviewed sample.

Our search yielded 275 articles. We used an active learning model to support screening and classification (Schoot et al. 2021; Burns et al. 2021; Schoot 2024). We selected a sample of 138 articles (50%) for initial screening. Then we filtered all articles by publication year, excluding studies that did not meet the citation count of ≥10 citations (up to 2015), 4 (2016–2018), 2 (2019–2020), and 1 (2021–2022), aiming to include the most relevant studies.

Among the included articles, we conducted a two-stage thematic analysis. First, we built a term map based on titles and abstracts, grouped terms by frequency, co-occurrence, and thematic clusters. We used the software VOSviewer 1.6.20, a tool for mapping the scientific landscape and identifying research gaps (Eck and Waltman 2023). Following, we cleaned, harmonized, and grouped author keywords from the reviewed articles, identifying the 20 most frequent terms and visualizing their use over time. To broaden our discussion, we also carried out a manual search on Google Scholar (“restoration project Atlantic Forest”) and compared key findings with those from Scopus.

In addition to peer-reviewed articles, we compiled a contextual set of documented restoration initiatives in the Atlantic Forest identified during the review process. This contextual component was analyzed separately from the peer-reviewed sample and was used to examine how restoration initiatives are publicly documented and whether these claims could be compared with findings reported in the peer-reviewed literature. Public reports, project websites, and institutional materials often reach practitioner and policy-facing audiences, with varying levels of detail on baseline conditions, methods, monitoring design, and results.

These materials were found through Google general and news searches using terms such as “nature restoration project” and “restoration project Atlantic Forest,” as well as through the Restor platform, institutional and NGO (Non-governmental organization) reports, project websites, and other public documents accessed during the literature review. The Restor platform was treated as a source of project records and contextual information. We also considered Brazilian projects cited in a complementary source (Castro 2024).

As a result, the documented cases represent an exploratory and opportunistic sample of relatively visible and well-documented initiatives. We manually filtered examples with sufficient public information for screening and classified them using the same geographic and thematic eligibility criteria applied to the review. When detailed findings were unavailable, these sources were used to characterize implementation contexts, governance and financing conditions, and publicly reported claims associated with restoration.

Peer-reviewed studies were examined for reported environmental and socioeconomic findings, as well as governance and financing conditions described in the literature. Environmental findings were grouped into carbon, biodiversity, water, and soil. Socioeconomic findings included livelihoods, costs, income generation, participation, and other reported social or economic effects. Governance and financing were treated separately as implementation conditions, including institutional arrangements, partnerships, incentives, and policy-related mechanisms. Documented initiatives were reviewed in parallel to characterize implementation contexts and publicly reported claims, while remaining analytically distinct from the synthesis of peer-reviewed findings. We then grouped recurring themes across sources to support the synthesis presented in the Results and Discussion, while maintaining a distinction between peer-reviewed findings and information drawn from documented initiatives.

## Results

### Profile of the Peer-reviewed Literature, Thematic Patterns, and Study Areas

After title and abstract screening, 100 articles underwent full-text review. With 3 unavailable and 10 that did not meet the inclusion criteria, we identified 87 articles for analysis (Supplementary File S1). We identified an increase in publications after 2014, with more recent studies surpassing earlier ones in number (Supplementary Figure S1).

**Fig. 2:**
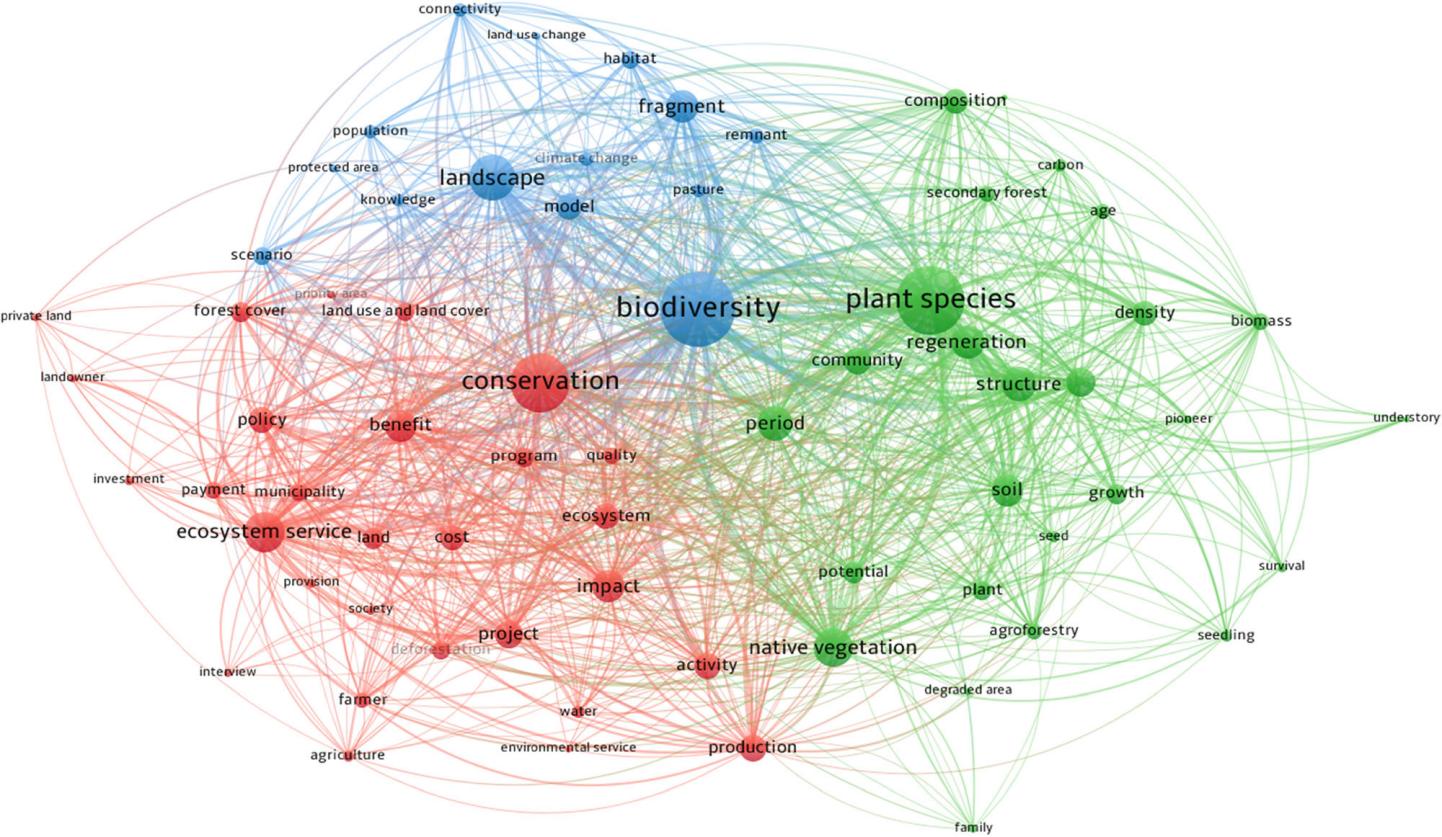
Term map based on titles and abstracts of articles, with colors representing thematic clusters

**Fig. 3:**
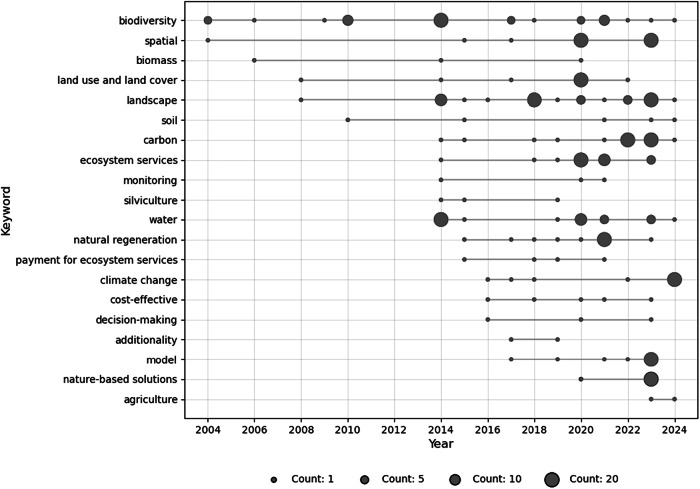
Number of author keyword occurrences per year of publication

As part of our thematic analysis, we built a term map (Fig. 2) that shows three main clusters: **landscape ecology** (blue), focusing on connectivity and habitat dynamics; **socioeconomic interactions** (red), related to ecosystem services and cost-benefit analysis; and **ecosystem composition and dynamics** (green), related to functional traits. *Biodiversity* appeared as a central cross-cutting term. Moreover, among the 20 most frequent keywords in the 87 articles (Fig. 3), *biodiversity* remained recurrent throughout the period, while *spatial* was already present in earlier years and became more frequent in recent years. *Carbon* and *water* also became more recurrent in the later part of the series, especially after 2014, while *climate change*, *model*, and *nature-based solutions* appeared mainly in the most recent years. *Monitoring* and *silviculture* appeared less frequently in recent years.

**Fig. 4:**
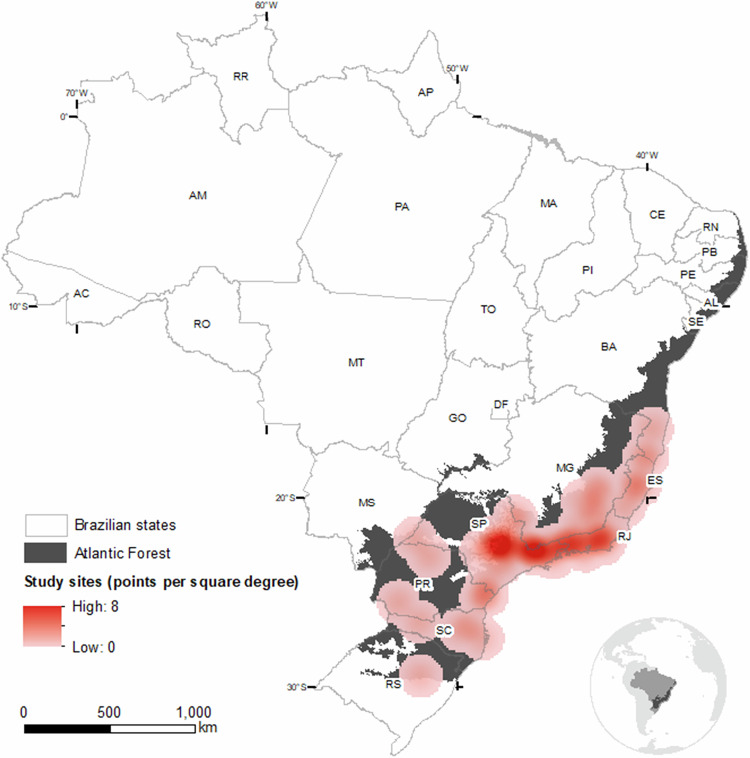
Density map of central study area points, excluding studies that covered the entire biome. Points were compiled from the reviewed articles, state and biome boundaries are from official cartographic datasets (IBGE 2019, 2022)

We found that these studies cover a wide range of states across Brazil (Fig. 4, with official boundaries of Brazilian states and the Atlantic Forest biome from IBGE 2019, 2022; Supplementary Fig. S2; Supplementary Table S1). Thirty-five studies were conducted in or included the state of São Paulo, likely due to its economic importance and institutional links through universities and government programs, as well as the Atlantic Forest Restoration Pact (Pinto et al. 2014) and SOS Mata Atlântica NGO (SOS Mata Atlântica 2026). Most cited articles cover the entire biome (Rodrigues et al. 2009; Banks-Leite et al. 2014; Tambosi et al. 2014; Strassburg et al. 2019). We identified a biogeographic bias, characterized by an overrepresentation of studies in Southeast Brazil and a lack of research from the Northeast, a pattern also reported in previous assessments (Schimetka et al. 2024).

### Documented Initiatives and Contextual Sources

As a contextual complement to the peer-reviewed sample, we compiled documented restoration initiatives from two complementary source streams (Supplementary Tables S2 and S3). The Restor platform and complementary gray literature sources were used to locate documented restoration initiatives and contextual information with sufficient detail for analysis. The platform contained more than 7000 records for Brazil, with heterogeneous levels of detail, verification, and implementation status. After applying filters for biome, project status, contact information, and area size greater than 100 ha, we retained 12 project records. These records included both private and public areas, with participation from companies, individuals, governments, non-governmental organizations, and communities. Most retained records were implemented in pasturelands and degraded forest areas (83%), while two were located in urban areas.

Additional documented initiatives identified through the broader contextual search included REGUA—Guapiaçu Ecological Reserve and IPÊ’s restoration programs. REGUA was also associated with peer-reviewed studies on spatial prioritization (Torres et al. 2019) and watershed-related opportunity costs (Osuna et al. 2014). In Pontal do Paranapanema, IPÊ’s long-term work, including the Corridors for Life initiative and agroforestry systems with MST (Landless Workers’ Movement) communities, was documented in studies linking endangered species conservation, forest corridor restoration, landholder engagement, and livelihood-related findings (Chazdon et al. 2020; Shennan-Farpón et al. 2022).

Two retained cases were also accompanied by additional case-study reports. Fazenda Bulcão, maintained by Instituto Terra, was identified in Restor records and in case-study documentation used as contextual information on implementation conditions and public reporting (Schweizer and Chazdon 2021). Mutirão Reflorestamento was also identified in Restor records and case-study documentation and was further assessed in the peer-reviewed literature as an urban restoration project in Rio de Janeiro, with reported socioeconomic effects and community perceptions based on focus groups and interviews in eight communities (Lemgruber et al. 2021; Chazdon et al. 2022).

### Environmental Evidence

The 63 studies reporting environmental evidence were synthesized into findings on carbon, biodiversity, water, and soil (Supplementary File S1; Supplementary Tables S4–S6). Most on-site assessments showed that carbon and biodiversity recovery often remained below levels observed in mature forests, particularly in naturally regenerated areas (Hopp et al. 2010; Shimamoto et al. 2014; Souza et al. 2016; Sansevero et al. 2017; Gomes et al. 2020; Matos et al. 2020b; Rosa et al. 2021; Coelho et al. 2022b; Vibrans et al. 2022; Arcanjo et al. 2024). Tree plantations and intensive silviculture were associated with faster carbon accumulation, but often at higher financial cost and with lower biodiversity gains (Ferez et al. 2015; Brancalion et al. 2019a, 2020, 2021; Pontes et al. 2019). Biodiversity tended to increase over time, although recovery remained strongly influenced by landscape connectivity and site management (Rolim et al. 2017; Gomes et al. 2020; Pyles et al. 2020; Coelho et al. 2022b, a).

At the landscape scale, increases in forest cover did not necessarily translate into full recovery of water regulation, soil protection, or related benefits, especially where fragmentation remained high and early-successional stands predominated (Ferraz et al. 2014). Recovery also varied with time since intervention, restoration pathway, previous land use, soil conditions, and landscape context. Some studies found slow or partial recovery even after decades. Second-growth forests after long-term eucalyptus plantation abandonment recovered only part of old-growth carbon stocks and species richness after 32 years (Coelho et al. 2022b). In naturally regenerated secondary forest fragments, aboveground carbon levels reached only about one-fifth of those in primary forests after three decades, while several biodiversity metrics recovered more strongly (Matos et al. 2020a). Carbon restoration costs also remained linked to intervention type, forest age, and site conditions, with total carbon stocks often remaining below those of reference forests during restoration trajectories (Brancalion et al. 2021). Previous land use and management intensity shaped recovery trajectories, with some secondary forests showing low biomass accumulation or simplified communities decades after abandonment (Robinson et al. 2015; Sansevero et al. 2017). Soil conditions and landscape context, including isolation from propagule sources, also influenced forest structure, diversity, and carbon recovery (Coelho et al. 2022a). In the reviewed sample, water-related evidence relied more often on modeled estimates of watershed services, erosion control, water quality, or restoration priorities than on long-term field measurements of streamflow or water quality (Osuna et al. 2014; Vettorazzi and Valente 2016; Viani et al. 2019; Saad et al. 2021). Soil evidence also showed that belowground recovery may not follow the pattern of aboveground biomass accumulation (Quartucci et al. 2023). Variation in restoration age, previous land use, metrics, and time frames limits direct comparison across studies and makes forest-cover gain an insufficient proxy for broader ecological recovery.

Among the reviewed articles, 22 applied models and analytical approaches, such as spatial prioritization, to guide restoration planning. They estimated potential gains and helped identify where restoration could be prioritized across landscapes and, in some cases, at the rural property level. Across this group, the main objectives were landscape connectivity, carbon storage, erosion control, ecological suitability, and cost-effectiveness (Stoms et al. 2004; Strassburg et al. 2016, 2019; Marjakangas et al. 2018; Lemos et al. 2021, 2023; Brancalion et al. 2021; Domingues et al. 2023). Some studies also estimated potential gains in water quality and soil retention based on variables such as topography, soil type, and erosion risk (Osuna et al. 2014; Strassburg et al. 2016; Vettorazzi and Valente 2016; Lemos et al. 2021; Saad et al. 2021; Valente et al. 2021; Lemos et al. 2023; Santos et al. 2023a). While most of these studies focused on landscape-scale scenarios, some also stressed prioritization at the rural property level to increase implementation feasibility (Benzeev et al. 2023). The reviewed model-based studies used spatial planning to align ecological goals with financial constraints, especially across landscape and property contexts. Few studies compared alternative modeling approaches or assessed whether mapped priorities were implemented and monitored after restoration began. Few studies tested whether mapped priorities were later implemented or monitored.

Across a small set of seven stakeholder and perception-based studies, biodiversity, water, and soil were often valued for their economic or symbolic meanings (Lemgruber et al. 2021). Water-related gains, such as spring recovery and flood mitigation, are especially relevant in local narratives (Lemgruber et al. 2021; Sales and Guedes-Bruni 2023). Restoration finance mechanisms are more likely to succeed when linked to visible, short-term benefits and complementary practices, such as erosion control or rural infrastructure improvements (Alarcon et al. 2017; Santos et al. 2020; Maioli et al. 2021). In the reviewed studies, local participation and acceptance were more often associated with strategies aligned with perceived priorities, practical needs, and visible short-term benefits.

### Social Evidence

Social evidence was less frequently reported, with only eight articles and a few documented initiatives presenting direct assessments. Few documented initiatives described direct social effects, mainly job creation, income generation, training, and participation in restoration activities. Agroforestry was the main context in which income generation was reported, especially through primary and secondary forest products, community participation, and support for smallholder farmers (Shennan-Farpón et al. 2022; Souza et al. 2016; IPÊ—Sistemas Agroflorestais). Additional income from payments for ecosystem services (PES) was also reported in restoration programs, especially through payments for watershed services to rural landowners (Santos et al. 2020). Social acceptance and landholder engagement were also reported as important conditions for long-term continuity (Lemgruber et al. 2021; Oliveira et al. 2021). One documented initiative involved inmates in restoration activities, combining income opportunities with training (Abreu et al. 2022). Across the reviewed sample, however, these social effects were reported heterogeneously and rarely through standardized indicators, which limited direct comparison across cases.

In addition to these direct social effects, socioeconomic factors associated with forest regrowth were also assessed. In this literature, land abandonment was the most recurrent factor, alongside population density, Gross Domestic Product, and proximity to transport infrastructure and urban areas (Molin et al. 2017). These studies help explain where regrowth occurs and the broader socioeconomic and land-use contexts in which forest recovery takes place (Baptista 2008), but they do not assess social outcomes in the same sense as, income, training, or participation.

Studies also reported perceived cultural and recreational values related to restoration, including landscape esthetics, recreation, tourism, religion, spirituality, and education (Brancalion et al. 2014). Some documented initiatives incorporated environmental education and ecotourism into broader restoration strategies (IPÊ – Semeando Água; Instituto Terra).

The social studies combined different types of evidence, including direct effects, regrowth drivers, and perceived cultural values, which limited the ability to compare them.

### Financial Evidence

Twenty-one studies assessed financial aspects of forest restoration, with a primary focus on opportunity costs. While plantations may store more carbon, second-growth forests were often associated with lower restoration costs under specific ecological and land-use conditions (Brancalion et al. 2021). Socioeconomic conditions and the cost-saving potential of natural regeneration were recurrent considerations in studies discussing restoration efficiency (Molin et al. 2018; Niemeyer et al. 2020). In model-based analyses, larger restoration allocations were estimated to benefit from economies of scale (Strassburg et al. 2019), while smallholder contexts were associated with lower opportunity costs when regeneration aligned with agricultural landscape goals (Osuna et al. 2014; Strassburg et al. 2016; Shennan-Farpón et al. 2022).

Financing mechanisms are widely discussed. The reviewed studies proposed adapting incentives for smallholders (Ruggiero et al. 2019; Richards et al. 2020; Lemos et al. 2023), including reallocating agricultural subsidies (Banks-Leite et al. 2014) and using water charges to fund PES (Viani et al. 2019). Reported PES payments reached up to US$30/ha annually in some cases (Santos et al. 2020), whereas restoration costs reported between 2014 and 2021 were substantially higher and varied widely with land use, location, and project scope (Osuna et al. 2014; Alarcon et al. 2017; Fiorini et al. 2020; Saad et al. 2021). These figures are not directly comparable, as they compare annual incentive payments with restoration implementation costs, but they indicate a significant financing gap.

Cost-benefit evaluations include water treatment savings and carbon markets. In the Guapi-Macacu region (Rio de Janeiro state), conservation proved more cost-effective than land-use change for water services (Osuna et al. 2014). Additionally, in abandoned pasturelands, carbon and sediment benefits might offset opportunity costs within 20 years (Strassburg et al. 2016). Municipalities facing more disasters tend to adopt PES, even if not directly aimed at risk reduction (Almeida et al. 2023).

The financial evidence reported in the reviewed sample focused mainly on implementation costs, cost drivers, and financing-related constraints, with fewer studies directly assessing financial outcomes. Lower-cost methods, such as nucleation, were estimated to cost about 30% less than high-diversity planting (Bechara et al. 2021). Site-specific risks, intervention type, and funding source were recurrent factors shaping these estimates, and fire risk was also identified as a relevant planning consideration (Guedes et al. 2020). Reported costs for active restoration typically range from US$5,000 to US$6,500/ha in the Atlantic Forest (Table S3), depending on logistics, labor, and site conditions. These high costs remained a barrier to large-scale implementation, given continued reliance on public funding and limited private investment under uncertain returns (Ermgassen and Löfqvist 2024). Some initiatives also framed restoration costs in terms of avoided losses, such as drought-related impacts, to build a stronger economic case (TNC—Reservatório Invisível).

In the reviewed sample, few studies reported financial estimates alongside measured ecological results, and cost information was rarely standardized across cases. Financial estimates could not be consistently linked to ecological performance.

### Governance-related Evidence

Governance-related conditions were described as influencing how restoration is organized, implemented, and sustained over time across landscapes (Chazdon et al. 2021). The theme was examined in 15 articles and a few documented initiatives, which we grouped into four categories: community-based arrangements, private landholder compliance and engagement, incentive-based mechanisms, and multi-level coordination. Across these categories, the reviewed studies most often linked governance-related conditions to participation, implementation feasibility, continuity over time, and coordination across actors and scales.

Community-based arrangements were described as important implementation conditions in several restoration cases. Studies on *Mutirão Reflorestamento* program (Rio de Janeiro state) and settlement-based experiences showed that restoration efforts were shaped by local organization, public support, and coordination with urban communities, local authorities, and smallholders (Lemgruber et al. 2021; Chazdon et al. 2022; Shennan-Farpón et al. 2022; Quartucci et al. 2023). In contexts marked by agrarian reform or transitions from traditional agricultural practices, participation depended in part on how well restoration strategies aligned with local institutions, livelihoods, and social realities (Gomes et al. 2020). Local participation was strongest when restoration strategies aligned with local institutions, livelihoods, and perceived barriers. However, these studies did not report governance outcomes consistently (Maioli et al. 2021).

On private lands, restoration was more often described in terms of legal compliance, landholder engagement, productive land use, and economic feasibility. Studies reported that private conservation units (RPPNs), technical support, and partnerships with NGOs were recurrent features in cases where restoration advanced across different landscapes (Banks-Leite et al. 2014; Niemeyer et al. 2020; Nascibem et al. 2023). These studies suggest that institutional support and lower implementation barriers helped make restoration more feasible on private lands. Certification schemes may also support restoration on private lands by linking market incentives to environmental compliance. For example, coffee certification standards have included zero-deforestation rules, minimum native vegetation requirements, and restoration plans for riparian and other sensitive areas, while certified farms in the Atlantic Forest were reported to restore more protected areas than non-certified farms (d’Albertas et al. 2023).

Incentive-based mechanisms, such as PES, were described with mixed implementation experiences that depended on local context and program design. In the Water Producer/PCJ program (São Paulo state), implementation was reported to be more feasible where conservation practices did not require major land-use change, although challenges remained despite available funding (Viani et al. 2019). Other studies reported that high costs, limited monitoring, and technical constraints affected implementation, especially in poorly planned projects (Santos et al. 2020). Land-use conflicts were also described in areas where restoration overlapped with agricultural or forestry interests, particularly in abandoned pasturelands, plantation expansion zones, and areas targeted for restoration (Strassburg et al. 2016; Cerullo et al. 2024). Some studies proposed multi-criteria approaches to help balance competing interests in these contexts (Vettorazzi and Valente 2016; Zwiener et al. 2017). Overall, this group of studies showed that incentives alone were often insufficient when restoration required major land-use change or when implementation and monitoring conditions were weak.

Several studies described multi-level coordination as part of restoration implementation. The Atlantic Forest Restoration Pact was presented as a national arrangement that connects actors across sectors and scales (Pinto et al. 2014). At the local level, the *Water Conservation* program in the municipality of Extrema (Minas Gerais state) was described as a municipal initiative involving landowners, civil society, and government actors in restoration-related action (Richards et al. 2015). These cases illustrate coordination structures associated with restoration implementation, especially in linking actors, scales, and institutional agendas, but the reviewed studies did not report standardized governance outcomes that would allow direct comparison across cases. They also noted recurring challenges, especially where institutional goals were not matched by landowner participation (Viani et al. 2019).

In the reviewed studies, restoration on private lands was more often reported when technical support, incentives, or institutional partnerships were present. Across the sampled articles, governance most often shaped restoration through participation, implementation feasibility, continuity over time, and coordination across actors and scales. At the same time, opportunity costs, weak enforcement, coordination gaps, and discontinuous funding were recurrent constraints discussed in the literature.

## Discussion

### What the Current Evidence Base can and Cannot Support

The Atlantic Forest literature is uneven across both outcomes and evidence types. Many studies document implementation, estimate potential benefits, or report ecological and social outcomes. Far fewer studies isolate restoration effects using baseline data, control or comparison sites, counterfactuals, or long-term monitoring. Direct field evidence was more frequent for carbon and biodiversity, more limited for soil, and scarce for hydrological and social outcomes. Reported gains, therefore, cannot always be attributed to restoration alone, especially when previous land use, landscape context, site selection, monitoring duration, and broader socioeconomic change are not accounted for. Aboveground carbon accumulation and some biodiversity gains were more often measured directly, especially in active or intensively managed systems. Soil carbon tended to recover more slowly, and water-related responses remained less consistently measured and harder to generalize across sites (Filoso et al. 2017; Mendes et al. 2019; Dib et al. 2023). Even where forest cover expands, many restored landscapes remain dominated by young or early-successional stands, limiting the recovery of functions associated with older forests. Restoration in the biome can produce measurable gains, but the pace and extent of recovery depend on intervention type, site history, landscape context, and time since implementation.

The review also showed that model-based studies are widely used in restoration planning, especially for spatial prioritization involving biodiversity, carbon, erosion control, water-related benefits, and costs (Strassburg et al. 2016; Ribeiro et al. 2020). The use of spatial prioritization is also consistent with broader calls to integrate forest restoration into land-use planning at larger scales (Hua et al. 2024). These studies identify priority areas and estimate potential gains under explicit assumptions, generally at scales larger than individual restoration sites. Their outputs, however, still need to be compared with implementation data, long-term field monitoring, baseline conditions, and matched control or comparison sites. Monitoring frameworks for the Atlantic Forest already provide ecological, socioeconomic, and management indicators that can support this step, while evidence syntheses and restoration comparisons also point to the need for explicit site-selection and comparison design (Viani et al. 2017; Reid et al. 2018). Property-level information on land tenure, access, costs, local institutions, and production systems is also needed to assess whether mapped priorities can be implemented. Current literature is stronger at indicating where restoration should be prioritized than at evaluating whether model predictions hold once restoration begins.

Social and economic evidence remains less standardized and less frequent. In the reviewed sample, direct social effects were reported mainly as jobs, income generation, training, participation, and PES-related incentives, with agroforestry appearing as the main setting in which livelihood gains were described. By contrast, broader issues such as inequality, tenure security, labor conditions, and the distribution of benefits and costs were much less consistently assessed. Financial evidence focused on opportunity costs, implementation costs, and incentive design, but was rarely reported alongside measured ecological performance. This makes it difficult to compare interventions by cost and by the relationship between cost and carbon, biodiversity, water, soil, or social effects. The main gap is the lack of studies that connect costs, outcomes, and feasibility within the same cases (Schimetka et al. 2024; Ermgassen and Löfqvist 2024).

### Governance and Policy Implications

Governance was rarely measured through standardized indicators. It appeared mainly through implementation conditions such as local participation, technical support, institutional coordination, incentive design, and the feasibility of acting on private lands. Across the reviewed cases, restoration was more often reported in contexts where these conditions were combined, especially when landholders had technical support, some financial backing, and clear institutional partners. Weak enforcement, discontinuous funding, opportunity costs, and poor coordination repeatedly appeared as constraints. Restoration depends on arrangements that connect legal obligations, local practice, and long-term follow-up (Chazdon et al. 2021).

The reviewed cases stress a policy gap between restoration priorities and implementation capacity. Restoration policies need to connect ecological priority with property-level information, land tenure, local production systems, and municipal capacity. On private lands, legal requirements remain central, but they rarely translate into restoration without technical assistance, simple monitoring procedures, and incentives aligned with smallholder and municipal constraints. Institutional support could include lower compliance transaction costs, technical assistance after implementation begins, stable local coordination, and finance linked to measurable indicators. Connections with water security, rural livelihoods, food-system resilience, and productive landscape planning can also improve local feasibility and long-term permanence (Rezende et al. 2018; Matthews et al. 2022; Lopes and Chiavari 2024).

The Atlantic Forest Restoration Pact and local programs such as Extrema’s water conservation efforts remain useful examples of coordination across scales. Multi-level coordination can connect actors, resources, and agendas that no single project can sustain on its own. Formal coordination did not always translate into landowner participation or sustained implementation. Future work and policy should therefore pay closer attention to whether institutional arrangements persist over time and whether they support the long-term permanence of restoration at the landscape level.

### Peer-reviewed and Gray Literature

The synthesis is based on the publication record and documented initiatives available to this review. This material captures how restoration has been studied and reported, and only partially covers the factors that shape restoration practice in the Atlantic Forest. Social effects, governance, finance, and hydrological responses are the clearest underrepresented dimensions, because they are often monitored less regularly, reported in less detail, or documented outside peer-reviewed outlets. Low frequency in our sample, therefore, reflects limits in documentation and reporting. The practical role of these dimensions requires separate assessment.

Among the additional sources explored, the Restor platform was useful for locating project records and contextual information. Its environmental layers are derived from global remotely sensed or modeled products (Hansen et al. 2013; Freire et al. 2016; Running et al. 2017; Running and Zhao 2019), which were used only as contextual information, since project outcome evidence required site-level monitoring, project-specific measurements, or clearly reported implementation results. Gray literature helped contextualize implementation settings, institutional arrangements, and publicly reported claims beyond the peer-reviewed record (Paez 2017). These materials depend on voluntary reporting and available documentation and are therefore likely to over-represent larger, better-structured, and more visible initiatives. Because the retained set was small, we used these sources only to describe implementation and reporting practices.

Some complementary peer-reviewed studies were mentioned if they helped interpret patterns already visible in the reviewed sample. For example, some studies expand the discussion of spatial planning by incorporating socioeconomic and institutional dimensions into restoration prioritization (Ribeiro et al. 2020), while others call for more operational monitoring frameworks that connect management, vegetation, and soil indicators over time (Viani et al. 2017; Mendes et al. 2019). Additional works revisit the trajectory of restoration practice in the biome and stress the need for closer alignment between legal instruments, policy implementation, and adaptive strategies to sustain restoration over time (Rodrigues et al. 2009; Rother et al. 2023). They helped identify where evidence is uneven or poorly integrated.

### Trends, Gaps, and Future Research

Research on Atlantic Forest restoration has grown substantially since the early 2000s, with a marked increase after 2014 and a broader spread of themes thereafter. Earlier studies in our sample focused more on natural regeneration, forest succession, landscape structure, and broader planning questions, while later studies devoted more space to ecosystem services, carbon, water, spatial prioritization, and cost analyses (Zupo et al. 2022). Landmark contributions in 2004 and 2009 already raised questions about scale, cost, and sociopolitical barriers (Rolim and Chiarello 2004; Stoms et al. 2004; Rodrigues et al. 2009), while the diversification visible after 2014 is consistent with the expansion of landscape-scale planning, ecosystem service framing, and climate-related agendas in the biome (Banks-Leite et al. 2014; Tambosi et al. 2014; Strassburg et al. 2016; Vettorazzi and Valente 2016; Brancalion et al. 2019a; Viani et al. 2019). Part of this shift may also reflect the wider attention given to drought, climate risk, and resilience in southeastern Brazil after 2014–2015 (Nobre et al. 2016).

The field has become broader and more operational, but integration across themes remains limited. The term map and keyword analysis suggest that landscape ecology, ecosystem dynamics, and socioeconomic themes are still often discussed in largely separate analytical spaces. Similar difficulties appear in the wider literature on restoration governance and interdisciplinarity, especially in efforts to connect ecological assessment, land-use planning, and institutional implementation within a single analytical frame (Thiemann et al. 2018; Fernandes et al. 2021). In our sample, this separation is visible in the limited overlap among soil, hydrology, finance, governance, and social distributional effects within the same cases.

The term map also shows limited connections among themes that matter for implementation. *Agriculture* and *water* rarely appeared together, suggesting that restoration is still not often discussed in direct relation to food systems and productive landscape resilience, although these links are likely to affect implementation and permanence (Matthews et al. 2022). A weak association between *soil* and *cost* points to another gap: the limited number of studies that connect soil recovery and erosion control to financial evaluation. Distant links between agroforestry and landscape-scale planning suggest that local productive arrangements and broader spatial strategies are still too often treated separately, even though implementation depends on both. Likewise, the limited connection between *biomass* or *carbon* terms and *period* framing is consistent with continued uncertainty around long-term carbon accumulation trajectories under different site conditions and restoration pathways (Arcanjo et al. 2024).

Long-term comparative studies across intervention types are still needed. Direct ecological monitoring needs to be combined more often with financial, social, and governance data in the same cases. Hydrological and soil responses also require more field-based work over longer time scales, as they remain less consistently documented than carbon and biodiversity. Research is still sparse in underrepresented regions, especially in the Northeastern portion of the biome. Model-guided planning also needs more comparison with implementation data, so that prioritization tools can be assessed by estimated gains, feasibility, continuity, land tenure, local institutions, and production systems. Complementary work outside the main sample supports this direction, especially studies calling for operational monitoring frameworks, stronger integration between restoration targets and implementation conditions, and closer translation between spatial planning and institutional realities (Viani et al. 2017; Mendes et al. 2019; Ribeiro et al. 2020; Pfeifer et al. 2022; Hernandez et al. 2024).

### Synthesis

In the Atlantic Forest, restoration is better framed as a long-term land-use strategy linked to deforestation control and the reduction of degradation. The reviewed evidence shows strong variation across interventions, landscapes, and social settings. Carbon and some biodiversity outcomes are more frequently measured, while soil, hydrological, social, financial, and governance-related effects are less consistently tracked. Model-based studies help identify where restoration may yield multiple gains or lower opportunity costs, but their results need to be read alongside implementation data, baseline conditions, monitoring design, and land-tenure constraints.

Peer-reviewed studies provide more comparable information on measured outcomes, whereas documented initiatives and gray literature add context on visible projects, partnerships, financing, and public claims. Across sources, the main limitation is the uneven reporting of baseline conditions, monitoring design, comparison sites, and long-term results. These gaps affect how restoration effects and implementation claims can be compared across cases.

### Recommendations

Table 2 translates the main reporting and monitoring gaps found in the review into practical steps for planning, implementing, monitoring, and reporting Atlantic Forest restoration. These steps focus on the minimum information needed to compare cases and check restoration claims: baseline conditions, project descriptors, monitoring duration, links between spatial priorities and implementation data, and support for connecting legal requirements with field practice.

**Table 2:** Practical recommendations for planning, monitoring, and reporting Atlantic Forest restoration

Recommended actions	Primary users	Minimum action or information
Establish baseline and comparison conditions	Researchers, practitioners, funders	Record previous land use, soil, landscape context, and socioeconomic setting before intervention. Use comparison sites when feasible.
Report basic project descriptors	Practitioners, platforms, public agencies	Include intervention type, area, land tenure, funding source, restoration age, implementation stage, and monitoring duration.
Monitor fewer indicators for longer periods	Practitioners, researchers, monitoring programs	Track indicators linked to each project’s goals, including vegetation, carbon, soil, water, and selected social outcomes when relevant.
Compare maps with implementation data	Modelers, planners, public agencies	Check spatial priorities against field monitoring, costs, access, land tenure, local institutions, and production systems.
Connect legal duties with finance and technical support	Public agencies, funders, NGOs	Pair restoration requirements with assistance, incentives, simple monitoring, and local coordination, especially on private lands.

## Supplementary information

Supplementary File S1

Supplementary File S2

## Data Availability

All data supporting the findings of this study are available within the paper and its Supplementary Information.
