# Supplementary material for: Restoration Contexts and Trends in the Brazilian Atlantic Forest: Evidence from Science and Practice
Source: Environ Manage. 2026 Jun 12;76(6):215. doi: 10.1007/s00267-026-02528-8 (PMC13263303; doi:10.1007/s00267-026-02528-8)
Supplement: Supplementary file 2 — Supplementary File S2 [file 267_2026_2528_MOESM2_ESM.docx]

**Supplementary material – Restoration contexts and trends in the Brazilian Atlantic Forest: Evidence from science and practice**

# Results

## Profile of the peer-reviewed literature, thematic patterns, and study areas


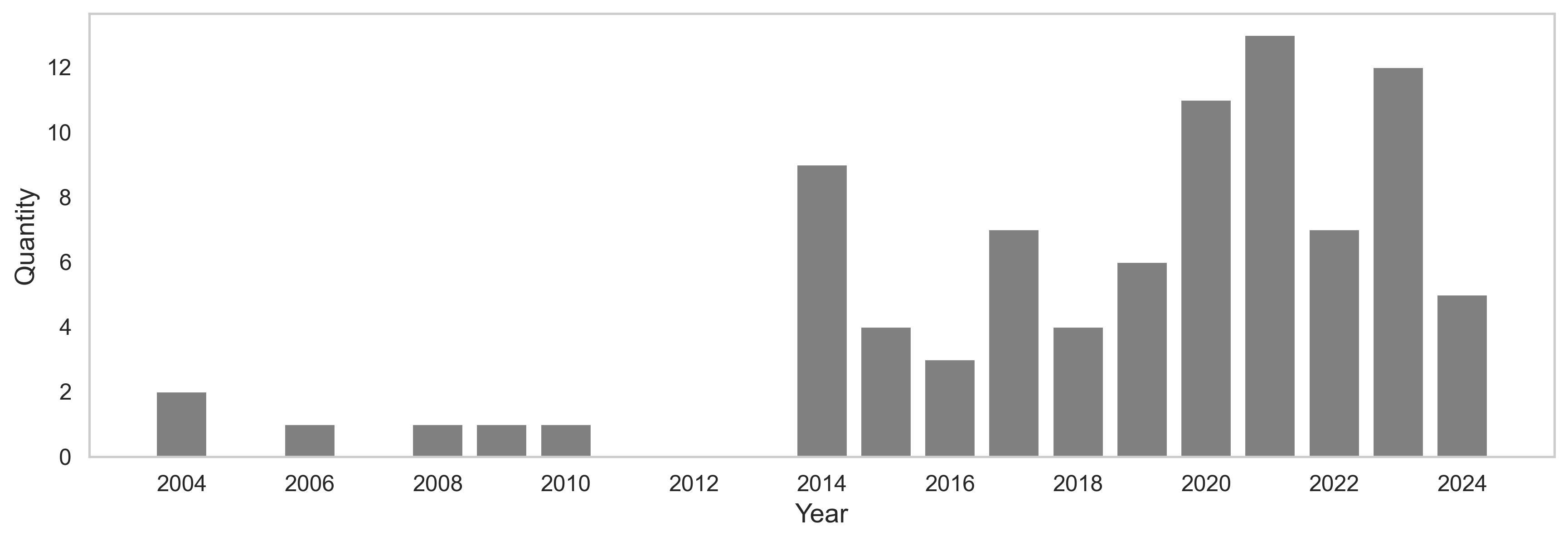


Figure S1 – Quantity of scientific articles per year of publication.


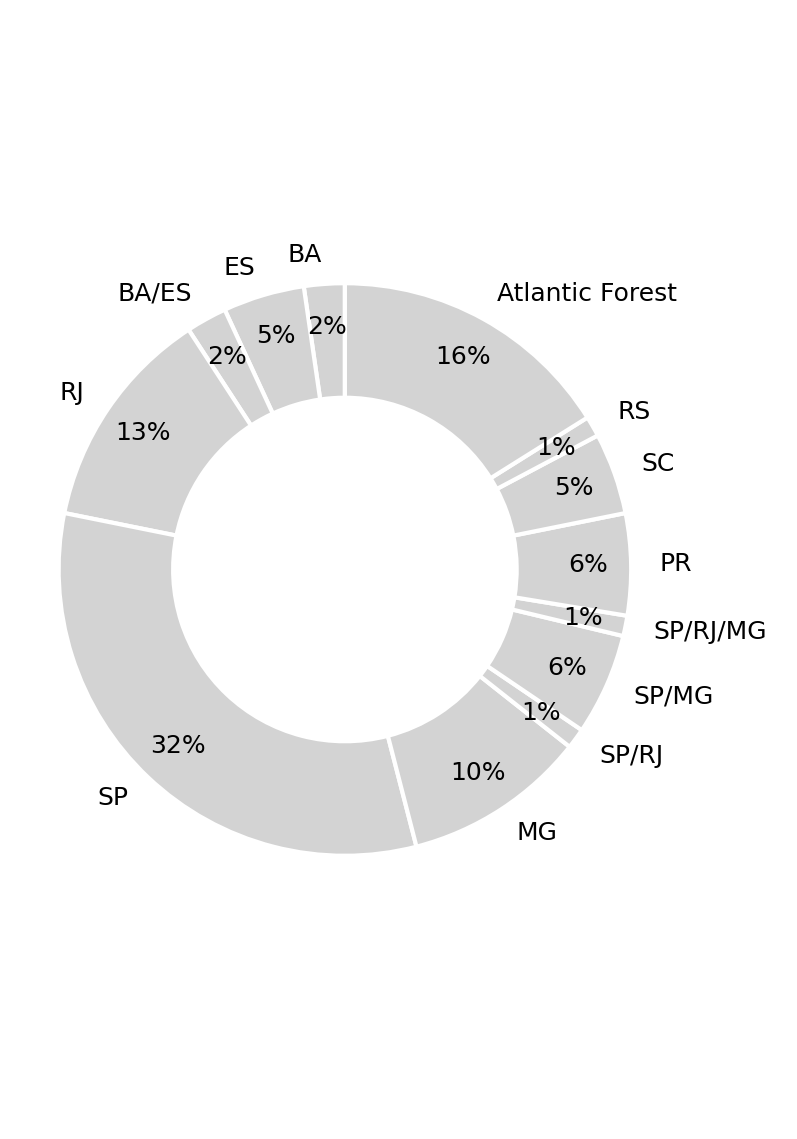


Figure S2 – Distribution of study areas per state and the entire biome.

Table S1 – Brazilian states' names and abbreviations

| State | Abbreviation |
| --- | --- |
| Acre | AC |
| Alagoas | AL |
| Amapá | AP |
| Amazonas | AM |
| Bahia | BA |
| Ceará | CE |
| Distrito Federal | DF |
| Espírito Santo | ES |
| Goiás | GO |
| Maranhão | MA |
| Mato Grosso | MT |
| Mato Grosso do Sul | MS |
| Minas Gerais | MG |
| Pará | PA |
| Paraíba | PB |
| Paraná | PR |
| Pernambuco | PE |
| Piauí | PI |
| Rio de Janeiro | RJ |
| Rio Grande do Norte | RN |
| Rio Grande do Sul | RS |
| Rondônia | RO |
| Roraima | RR |
| Santa Catarina | SC |
| São Paulo | SP |
| Sergipe | SE |
| Tocantins | TO |

##

## Documented initiatives and contextual sources

Table S2 – Reforestation sites in the Atlantic Forest in the Restor platform (https://restor.eco/)

| **Site name** | **Website/Documentation** | **Location** | **Area size (ha)** | **Start** | **Type of intervention** | **Pre/post-intervention land cover** | **Site management/**  **ownership** |
| --- | --- | --- | --- | --- | --- | --- | --- |
| Golden Lion Tamarin Association | https://micoleao.org.br/ | Silva Jardim (RJ) | 101 | 2021 | Active restoration | Deforested land/  Natural forest | NGO/Private |
| 01 – Fazenda Enseada – Tropical Flora | https://futuroflorestal.com.br/ | Garça (SP) | 120 | 2003 | Agroforestry | - | - |
| B2B42017RE | https://www.suzano.com.br/  sustentabilidade/planeta/meio-ambiente | Vereda (BA) | 150 | 2017 | Passive natural regeneration | Grazing land/  Multiple | - |
| Mutirão de Reflorestar/Brazil case study | https://storymaps.arcgis.com/  stories/7afa6040cd4e46b4872  0e280b7238434/  Chazdon et al. (2022); Lemgruber et al. (2021) | RJ | 167 | 2022 | Active restoration | Degraded forest/  Natural forest | Corporate/Private |
| A Hidden Paradise in a Troubled world | - | Ubaíra (BA) | 178 | 2021 | Active restoration | Deforested land/  Natural forest | - |
| Fazenda Bom Pastor | https://www.amap-brazil.org/pt | Ilhéus (BA) | 187 | 2018 | Active restoration | Grazing land/  Natural forest | - |
| RPPN Fazenda Bulcão | https://institutoterra.org/  Schweizer and Chazdon (2021) | Aimorés (MG) | 710 | 1998 | Active restoration | Grazing land/  Natural forest | Individual/Private |
| Parque Estadual Serra da Baitaca | https://amo-baitaca.wixsite.com/  2021 | Quatro Barras (PR) | 3,009 | 1994 | Assisted natural regeneration | - | - |
| Reserva Ambiental Nascentes do Rio Açungui | https://nascentes-do-rio-acungui.ueniweb.com/ | Campo Largo (PR) | 3,492 | 2002 | Active restoration | Degraded forest/  Grassland | NGO/Private |
| Fresco \| Projeto Pitanga | - | Itanhaém (SP) | 6,567 | 2024 | Active restoration | - | - |
| Parque das Neblinas | https://institutoterra.org/ | Mogi das Cruzes (SP) | 6,923 | 2022 | Assisted natural regeneration | Deforested land/  Natural forest | - |
| Caramuru-Paraguaçu reserve of the Pataxó HãHãHãe indigenous community | https://www.amap-brazil.org/pt/restoration | Itaju do Col. (BA) | 54,281 | 2023 | Agroforestry | Grazing land/Arid | Other |

Table S3 – Main characteristics and initiative-reported activities, outputs, and claims for selected Atlantic Forest restoration initiatives (Google; Castro, 2024)

| **Initiative** | **Website/Source/Documentation** | **Location** | **Area size** | **Start/**  **duration** | **Main initiative-reported activities, outputs, and claims** |
| --- | --- | --- | --- | --- | --- |
| Apremavi | https://apremavi.org.br/ | SC and PR | 640 ha in different municipalities | 1987 | 316 ha transformed into an RPPN (Private Reserve of Natural Heritage).  Participant perception from the ***Planejamento de Paisagens*** (Landscapes Planning) **p**rogram: the emergence of springs and the presence of trees made summer temperatures cooler and protected from frost in winter (Campanili 2018). |
| IPÊ – *Instituto de Pesquisas Ecológicas* (Institute for Ecological Research) | https://www.ipe.org.br/projetos  Shennan-Farpón et al. (2022) | Eight municipalities in the Cantareira System (SP and MG) | - | - | ***Semeando Água*** (Sowing Water) program: seeks to increase water security in the Cantareira System through forest restoration, promoting sustainable, productive systems, environmental education, and public policies. |
|  |  | Pontal do Paranapanema (SP) | - | 2001 | ***Corredores de Vida*** (Life Corridors) program: 6 million trees have already been planted to connect landscapes in the Atlantic Forest.  **Sistemas *Agroflorestais*** (Agroforestry Systems) program: promotes food diversity and income generation for rural producers. |
| *Iniciativa Verde* (Green Initiative) | https://www.iniciativaverde.org.br/ | SP, RJ, MG, RS, PR and BA | - | 2007 | It engages public, private, institutional, governmental, and community actors in various projects that integrate climate change, landscape restoration, and rural sanitation. |
| *Instituto Terra* (Earth Institute)/ RPPN Fazenda Bulcão | https://institutoterra.org/  Schweizer and Chazdon (2021) | Aimorés (MG), working in the Rio Doce River Basin (MG and ES) | 709.8 ha, 608.6 ha recognized as RPPN Fazenda Bulcão; another 344 acquired in 2022 | 1998 (institute creation)  1999 (first planting) | Nearly 3 million tree seedlings were planted for environmental recovery, seedling production, environmental education, and ecotourism activities.  Reported restoration costs of approximately R$36,000/ha (around US$6,500 at an exchange rate of 1 USD = 5.57 BRL) – from 2001 to 2023, with monetary correction for 2024. |
| Iracambi NGO | https://iracambi.com/ | Serra do Brigadeiro region (MG) | - | 2000 | It supports local schools and communities in planting 1 million native trees by 2030, of which 250,000 have already been planted. Engages with the mining industry’s interest, which has invested in social development projects.  Since 2015, following farmers' demand, it has worked with the Forests4Water project, which focuses on reforesting water catchment areas, especially on slopes, to stabilize soils and protect water supply. The process for selecting areas evaluates topography, soil type, vegetation cover, and water quality. |
| Olhos d’Água Farm | https://agendagotsch.com/en/ernst-gotsch/ | Piraí do Norte (BA) | ~500 ha | 1980s | Agroforestry management resulted in soil recovery from erosion, stream flow restoration, increased farm rainfall, and impacts on the surrounding microclimate (Fellet and Lima 2021; Kettley 2024). |
| re.green | https://re.green/ | Piraricaba (SP): office, seedling nursery  BA: Atlantic Forest projects located | 974 ha restored and 1,811 ha plantable, part of which is already in the restoration process | 2021 | It aims to capture 15 million tons of carbon per year (restoring 1 million hectares in the Amazon and Atlantic Forest) while conserving biodiversity, promoting sustainable management of forest products, and empowering local communities. |
| REGUA – *Reserva Ecológica do Guapiaçu* (Guapiaçu Ecological Reserve) | https://www.regua.org.br/  https://news.mongabay.com/2023/03/restoration-turns-pastures-into-wildlife-haven-in-brazils-atlantic-forest/  Torres et al. (2019); Osuna et al. (2014) | Guapiaçu River Basin (RJ) | 12,000 ha for conservation | 2006 | After planting 750,000 trees over the last two decades, it shelters nearly 500 bird species and hundreds of tree species. The organization has also acquired about 100 neighboring properties without agricultural potential. It promotes active restoration, with the per-hectare restoration cost estimated at US$5,000-US$6,000. |
| SOS Mata Atlântica – *Centro de Experimentos Florestais* (Center for Forest Experiments) | https://www.sosma.org.br/iniciativas/centro-de-experimentos-florestais | Itu (SP) | 366 ha restored | 2007 | It focuses on site prospecting, seedling production, planting techniques, monitoring, and stakeholder collaboration. Reports indicate the resurgence of two springs, a 5% increase in surface water, a 20% increase in groundwater, and a more than 150% increase in bird species. |
| Symbiosis | https://symbiosis.com.br/ | Porto Seguro (BA) | 1,500 ha recovered, 818 ha of forest planted for production, more than 1.3 million native trees | 2008 | In addition to silviculture, it acts directly in environmental recovery: it reforests degraded areas, restores the biodiversity of local flora and fauna, conserves the soil, and increases water production in springs. It also reclaims land for socioeconomic improvements and accounts for approximately 6.7 tons of CO_2_ fixed annually. |
| TNC - The Nature Conservancy Brazil | ***Conservador da Mantiqueira*** (Mantiqueira Conservator): https://conservadordamantiqueira.org | Mantiqueira region (RJ, MG and SP) | 1,5 million ha across 425 municipalities | 2016 | Collective strengthening through public policies, technical training, and governance networks. It seeks to channel resources and support municipalities in creating laws and promoting the restoration agenda with rural landowners and/or farmers. |
|  | ***Reservatório Invisível*** (Invisible Reservoir): https://www.tnc.org.br/conecte-se/comunicacao/artigos-e-estudos/reservatorio-invisivel/ | Cantareira System (SP and MG) | 32,085 ha | Study simulating scenarios in the period 1988-2018 | It identified priority areas considering wetlands, infiltration areas, and the potential for nebular capture.  Assessment of restoration costs and economic valuation of the impact of the 2014-2015 drought. |
|  | ***Restaura Brasil*** (Restore Brazil): https://www.tnc.org.br/o-que-fazemos/nossas-iniciativas/restaura-brasil/ | Atlantic Forest | 106 thousand ha restored (including Amazon and Cerrado savanna biomes) | 2001 | Implementing innovative techniques and structuring actions in the restoration chain, involvement with public policies, strengthening technical capacity, community collaboration, and collaboration with research partners. |

## Environmental evidence

Table S4 presents the main reported findings from empirical studies based on field measurements, organized by environmental aspect: carbon, biodiversity, water, and soil. To improve interpretability, we assigned each statement a finding type that reflects its primary role in the reviewed literature, such as an observed pattern, a management-related effect, an associated condition, an indicator, or a research gap.

Table S4 – Main reported findings from empirical studies based on field measurements

| **Environmental aspect** | **Main reported findings** | **Finding type** | **References** |
| --- | --- | --- | --- |
| Carbon | 1. Regenerated forests accumulate carbon more slowly and show lower carbon recovery than mature forests, even after decades. Re-clearance can be an additional undermining. | Observed pattern | (Shimamoto et al. 2014; Sansevero et al. 2017; Matos et al. 2020; Rosa et al. 2021; Zambiazi et al. 2021; Coelho et al. 2022b; Piffer et al. 2022; Vibrans et al. 2022; Arcanjo et al. 2024) |
|  | 2. Intensive silviculture or tree plantations can significantly increase biomass accumulation, especially at initial stages, but may be costly. | Management effect or trade-off | (Ferez et al. 2015; Brancalion et al. 2019, 2020, 2021; Pontes et al. 2019) |
|  | 3. Carbon stock is affected by site history, management intensity, and soil fertility. Soil carbon recovery may be slower than aboveground. | Associated condition | (Robinson et al. 2015; d’Albertas et al. 2018; Coelho et al. 2022a; Quartucci et al. 2023; Valente et al. 2023) |
| Biodiversity | 4. Species richness and diversity generally increase with time but may remain below that of mature forests. Re-clearance can be an additional undermining. | Observed pattern | (Hopp et al. 2010; Souza et al. 2016; Sansevero et al. 2017; Gomes et al. 2020; Matos et al. 2020; Rosa et al. 2021) |
|  | 5. Managed or planted systems can support diverse communities, especially when well-designed. | Management effect or trade-off | (Pontes et al. 2019; Brancalion et al. 2020; Bechara et al. 2021) |
|  | 6. Landscape context (e.g., proximity to other forests) and prior management influence biodiversity recovery. | Associated condition | (Robinson et al. 2015; Rolim et al. 2017; Sansevero et al. 2017; Coelho et al. 2022a) |
|  | 7. Specific taxa (e.g., butterflies, beetles) are useful indicators of forest structure and age. | Indicator or predictor | (Hopp et al. 2010; Sant’Anna et al. 2014) |
|  | 8. Some factors, such as vegetation indices, topography, soil properties, and rainfall interception, can be biodiversity predictors. | Indicator or predictor | (Gardon et al. 2020; Almeida et al. 2021; Rodrigues et al. 2021; Demetrio et al. 2024) |
|  | 9. Restoration increases species diversity, but prior management and isolation negatively affect it. | Observed pattern | (Rolim et al. 2017; Gomes et al. 2020; Pyles et al. 2020; Coelho et al. 2022b, a) |
| Water | 10. Forest restoration can improve water cycle functions like rainfall interception and vapor flux. | Observed pattern | (Gardon et al. 2020; Demetrio et al. 2024; Hollunder et al. 2024) |
|  | 11. Microclimatic factors (e.g., topography, drought refuge areas) are critical for water-related outcomes. | Associated condition | (Hollunder et al. 2024) |
|  | 12. Water quality improvement is an effective restoration strategy. | Management effect or trade-off | (Fiorini et al. 2020) |
| Water | 13. There is a need for better hydrological monitoring to assess water-related impacts. | Research gap | (Viani et al. 2019) |
| Soil | 14. Soil carbon recovery may not align with aboveground biomass accumulation. | Observed pattern | (Ferez et al. 2015; Quartucci et al. 2023) |
| Soil | 15. Soil properties (e.g., fertility, texture, and organic matter) influence restoration success. | Associated condition | (Robinson et al. 2015; Rodrigues et al. 2021; Demetrio et al. 2024) |
|  | 16. Past land use and soil conditions can limit restoration success, often requiring active interventions to support recovery. | Associated condition | (Ferretti and Britez 2006; Sansevero et al. 2017; d’Albertas et al. 2018; Coelho et al. 2022a) |

Table S5 summarizes the main reported findings from model-based analyses. We also assigned each statement a finding type according to its main role in the reviewed literature. These categories distinguish modeled planning implications, modeled conditions, indicators or predictors, modeled patterns, and research gaps.

Table S5 – Main reported findings from model-based analyses

| **Environmental aspect** | **Main reported findings** | **Finding type** | **References** |
| --- | --- | --- | --- |
| Carbon | 1. Strategic allocation and optimization models can improve restoration outcomes by simulating carbon sequestration while minimizing costs. | Modeled planning implication | (Strassburg et al. 2016, 2019; Lemos et al. 2021, 2023; Brancalion et al. 2021) |
|  | 2. Landscape features and proximity to remnants influence carbon recovery through natural regeneration. | Modeled condition | (Molin et al. 2018) |
| Biodiversity | 3. Restoration planning often uses habitat suitability, extinction risk reduction, and species-area curves. | Indicator or predictor | (Strassburg et al. 2016, 2019; Niemeyer et al. 2020; Cerullo et al. 2024; Shennan-Farpón et al. 2024) |
|  | 4. Species with gap-crossing capabilities benefit from landscape connectivity. | Modeled pattern | (Vettorazzi and Valente 2016; Zwiener et al. 2017; Viani et al. 2018) |
|  | 5. Maintaining a sufficient amount of native habitat is essential to supporting biodiversity and preventing rapid species loss, particularly in fragmented landscapes. | Modeled condition | (Banks-Leite et al. 2014; Newmark et al. 2017) |
|  | 6. Spatially explicit and optimized restoration planning supports biodiversity by improving habitat connectivity, representation, and ecological interactions while guiding efficient allocation of efforts and resources. | Modeled planning implication | (Stoms et al. 2004; Marjakangas et al. 2018; Lemos et al. 2021, 2023; Domingues et al. 2023) |
| Water | 7. Strategically planned restoration, aiming for connectivity, reverse degradation, and guided by the abiotic physical environment, can mitigate impacts on surface water quality and quantity – infiltration, groundwater recharge, and baseflow. | Modeled planning implication | (Osuna et al. 2014; Santos et al. 2023; Valente et al. 2021; Vettorazzi & Valente 2016; Possantti et al. 2023; TNC – Reservatório Invisível) |
|  | 8. Models must account for runoff dynamics and farm-scale outcomes to guide water-related decisions. | Research gap | (Possantti et al. 2023) |
| Soil | 9. Restoration can reduce soil erosion and sediment exports, especially when combining soil conservation practices. | Modeled planning implication | (Strassburg et al. 2016; Vettorazzi and Valente 2016; Lemos et al. 2021, 2023; Saad et al. 2021) |
|  | 10. Soil type and degradation risk are key in determining restoration priorities and biophysical suitability, but topography effects can overcome their importance. | Modeled condition | (Osuna et al. 2014; Valente et al. 2021; Possantti et al. 2023) |

Table S6 summarizes the main reported findings from perception-based studies and initiative-reported sources. To clarify the role of each statement, we also classified findings by type as perceived patterns, perceived outcomes, reported goals, associated conditions, and implementation needs.

Table S6 – Main reported findings from perception-based studies and initiative-reported sources

| **Environmental aspect** | **Main reported findings** | **Finding type** | **References** |
| --- | --- | --- | --- |
| Biodiversity | 1. Biodiversity and ecological processes become more evident a few years after restoration and tend to persist. | Perceived pattern | (Oliveira et al. 2021) |
|  | 2. Perceptions of biodiversity gains align with community engagement and restoration goals. | Associated condition | (Lemgruber et al. 2021; Maioli et al. 2021) |
| Water | 3. Water ecosystem functions, source recovery, and flood risk management are important goals in restoration strategies. | Reported goal | (Lemgruber et al. 2021; Sales & Guedes-Bruni 2023; Apremavi; IPÊ – Semeando Água; Olhos d'Água Farm; SOS Mata Atlântica – Centro de Experimentos Florestais; Symbiosis) |
|  | 4. Restoration is perceived to improve water quality. | Perceived outcome | (Brancalion et al. 2014; Maioli et al. 2021; Iracambi NGO) |
|  | 5. Effective forest restoration requires complementary actions and farmer engagement to address broader challenges such as erosion, pollution, and rural infrastructure, especially in water-stressed regions. | Implementation need | (Alarcon et al. 2017; Santos et al. 2020) |
| Soil | 6. Soil erosion control and land readjustment are necessary and recognized complementary actions for restoration. | Implementation need | (Alarcon et al. 2017; Santos et al. 2020; Maioli et al. 2021) |

# References

Alarcon GG, Fantini AC, Salvador CH, Farley J (2017) Additionality is in detail: Farmers’ choices regarding payment for ecosystem services programs in the Atlantic forest, Brazil. J Rural Stud 54:177–186. https://doi.org/10.1016/j.jrurstud.2017.06.008

Almeida DRAD, Broadbent EN, Ferreira MP, et al (2021) Monitoring restored tropical forest diversity and structure through UAV-borne hyperspectral and lidar fusion. Remote Sens Environ 264:. https://doi.org/10.1016/j.rse.2021.112582

Arcanjo FA, Lemos GG, Tartari LG, Torezan JMD (2024) Low predictability in aboveground biomass accumulation in Brazilian semi-deciduous seasonal Atlantic Forest restoration sites. Restor Ecol 32:. https://doi.org/10.1111/rec.14005

Banks-Leite C, Pardini R, Tambosi LR, et al (2014) Using ecological thresholds to evaluate the costs and benefits of set-asides in a biodiversity hotspot. Science 345:1041–1045. https://doi.org/10.1126/science.1255768

Bechara FC, Trentin BE, Engel VL, et al (2021) Performance and cost of applied nucleation versus high-diversity plantations for tropical forest restoration. For Ecol Manage 491:. https://doi.org/10.1016/j.foreco.2021.119088

Brancalion PHS, Amazonas NT, Chazdon RL, et al (2020) Exotic eucalypts: From demonized trees to allies of tropical forest restoration? J Appl Ecol 57:55–66. https://doi.org/10.1111/1365-2664.13513

Brancalion PHS, Campoe O, Mendes JCT, et al (2019) Intensive silviculture enhances biomass accumulation and tree diversity recovery in tropical forest restoration. Ecol Appl 29:. https://doi.org/10.1002/eap.1847

Brancalion PHS, Cardozo IV, Camatta A, et al (2014) Cultural ecosystem services and popular perceptions of the benefits of an ecological restoration project in the Brazilian Atlantic Forest. Restor Ecol 22:65–71. https://doi.org/10.1111/rec.12025

Brancalion PHS, Guillemot J, César RG, et al (2021) The cost of restoring carbon stocks in Brazil’s Atlantic Forest. Land Degradation & Development 32:830–841. https://doi.org/10.1002/ldr.3764

Campanili M (2018) Apremavi 30 years, 30 causes. Apremavi

Castro CDM (2024) The Tree Campaign: We Want Shade and Fresh Water. BEI EDITORA, São Paulo, SP

Cerullo G, Worthington T, Brancalion PHS, et al (2024) Conflicts and opportunities for commercial tree plantation expansion and biodiversity restoration across Brazil. Global Change Biol 30:. https://doi.org/10.1111/gcb.17208

Chazdon RL, Park H, Tedesco A (2022) Case #9: Mutirão Reflorestamento: a joint effort to restore forests in Rio de Janeiro, Brazil. ETH Zurich; Crowther Lab

Coelho AJP, Matos FAR, Villa PM, et al (2022a) Multiple drivers influence tree species diversity and above-ground carbon stock in second-growth Atlantic forests: Implications for the passive restoration. J Environ Manage 318:. https://doi.org/10.1016/j.jenvman.2022.115588

Coelho AJP, Villa PM, Matos FAR, et al (2022b) Atlantic Forest recovery after long-term eucalyptus plantations: The role of zoochoric and shade-tolerant tree species on carbon stock. For Ecol Manage 503:. https://doi.org/10.1016/j.foreco.2021.119789

d’Albertas F, Costa K, Romitelli I, et al (2018) Lack of evidence of edge age and additive edge effects on carbon stocks in a tropical forest. For Ecol Manage 407:57–65. https://doi.org/10.1016/j.foreco.2017.09.042

Demetrio WC, Brown GG, Pupin B, et al (2024) Soil macrofauna and water-related functions in patches of regenerating Atlantic Forest in Brazil. Pedobiologia 103:. https://doi.org/10.1016/j.pedobi.2024.150944

Domingues GF, Hughes FM, Santos AG dos, et al (2023) Designing an optimized landscape restoration with spatially interdependent non-linear models. Science of The Total Environment 873:162299. https://doi.org/10.1016/j.scitotenv.2023.162299

Fellet J, Lima F (2021) Ernst Götsch: the Swiss farmer who teaches how to “plant water” in Bahia. In: BBC News Brasil. https://www.bbc.com/portuguese/brasil-59269706. Accessed 2 Sept 2024

Ferez APC, Campoe OC, Mendes JCT, Stape JL (2015) Silvicultural opportunities for increasing carbon stock in restoration of Atlantic forests in Brazil. For Ecol Manage 350:40–45. https://doi.org/10.1016/j.foreco.2015.04.015

Ferretti AR, Britez RM de (2006) Ecological restoration, carbon sequestration and biodiversity conservation: The experience of the Society for Wildlife Research and Environmental Education (SPVS) in the Atlantic Rain Forest of Southern Brazil. J Nat Conserv 14:249–259. https://doi.org/10.1016/j.jnc.2006.04.006

Fiorini ACO, Mullally C, Swisher M, Putz FE (2020) Forest cover effects of payments for ecosystem services: Evidence from an impact evaluation in Brazil. Ecol Econ 169:. https://doi.org/10.1016/j.ecolecon.2019.106522

Gardon FR, Toledo RMD, Brentan BM, Santos RFD (2020) Rainfall interception and plant community in young forest restorations. Ecol Indic 109:. https://doi.org/10.1016/j.ecolind.2019.105779

Gomes EPC, Sugiyama M, Oliveira-Junior CJF de, et al (2020) Post-agricultural succession in the fallow swiddens of Southeastern Brazil. For Ecol Manage 475:. https://doi.org/10.1016/j.foreco.2020.118398

Hollunder RK, Garbin ML, Scarano FR, et al (2024) Vapor pressure deficit drives the mortality of understorey woody plants during drought recovery in the Atlantic Forest. J Veg Sci 35:. https://doi.org/10.1111/jvs.13222

Hopp PW, Ottermanns R, Caron E, et al (2010) Recovery of litter inhabiting beetle assemblages during forest regeneration in the Atlantic forest of Southern Brazil. Insect Conserv Diversity 3:103–113. https://doi.org/10.1111/j.1752-4598.2010.00078.x

Kettley A (2024) What soil is worth: A cost-benefit framework analysis of syntropic farming. Independent Study Project (ISP) Collection

Lemgruber LS, Maioli V, Latawiec AE, et al (2021) Socioeconomic impacts of urban restoration in the Atlantic Forest, Brazil. Urban For Urban Greening 64:. https://doi.org/10.1016/j.ufug.2021.127271

Lemos CMG, Andrade PR, Rodrigues RR, et al (2021) Combining regional to local restoration goals in the Brazilian Atlantic forest. Reg Environ Change 21:. https://doi.org/10.1007/s10113-021-01792-0

Lemos CMG, Beyer HL, Runting RK, et al (2023) Multicriteria optimization to develop cost-effective pes-schemes to restore multiple environmental benefits in the Brazilian Atlantic forest. Ecosyst Serv 60:. https://doi.org/10.1016/j.ecoser.2023.101515

Maioli V, Monteiro LM, Tubenchlak F, et al (2021) Local Perception in Forest Landscape Restoration Planning: A Case Study From the Brazilian Atlantic Forest. Front ecol evol 9:. https://doi.org/10.3389/fevo.2021.612789

Marjakangas E-L, Genes L, Pires MM, et al (2018) Estimating interaction credit for trophic rewilding in tropical forests. Philos Trans R Soc B Biol Sci 373:. https://doi.org/10.1098/rstb.2017.0435

Matos PS, Fonte SJ, Lima SS, et al (2020) Linkages among soil properties and litter quality in agroforestry systems of Southeastern Brazil. Sustainability 12:1–22. https://doi.org/10.3390/su12229752

Molin PG, Chazdon R, Ferraz SF de B, Brancalion PHS (2018) A landscape approach for cost-effective large-scale forest restoration. Journal of Applied Ecology 55:2767–2778. https://doi.org/10.1111/1365-2664.13263

Newmark WD, Jenkins CN, Pimm SL, et al (2017) Targeted habitat restoration can reduce extinction rates in fragmented forests. Proc Natl Acad Sci U S A 114:9635–9640. https://doi.org/10.1073/pnas.1705834114

Niemeyer J, Barros FSM, Silva DS, et al (2020) Planning forest restoration within private land holdings with conservation co-benefits at the landscape scale. Sci Total Environ 717:. https://doi.org/10.1016/j.scitotenv.2019.135262

Oliveira RE de, Engel VL, Loiola P de P, et al (2021) Top 10 indicators for evaluating restoration trajectories in the Brazilian Atlantic Forest. Ecological Indicators 127:107652. https://doi.org/10.1016/j.ecolind.2021.107652

Osuna VR, Börner J, Nehren U, et al (2014) Priority areas for watershed service conservation in the Guapi-Macacu region of Rio de Janeiro, Atlantic Forest, Brazil. Ecol Processes 3:. https://doi.org/10.1186/s13717-014-0016-7

Piffer PR, Calaboni A, Rosa MR, et al (2022) Ephemeral forest regeneration limits carbon sequestration potential in the Brazilian Atlantic Forest. Global Change Biol 28:630–643. https://doi.org/10.1111/gcb.15944

Pontes DMF, Engel VL, Parrotta JA (2019) Forest structure,wood standing stock, and tree biomass in different restoration systems in the Brazilian Atlantic forest. Forests 10:. https://doi.org/10.3390/f10070588

Possantti I, Barbedo R, Kronbauer M, et al (2023) A comprehensive strategy for modeling watershed restoration priority areas under epistemic uncertainty: A case study in the Atlantic Forest, Brazil. Journal of Hydrology 617:129003. https://doi.org/10.1016/j.jhydrol.2022.129003

Pyles MV, Magnago LFS, Borges ER, et al (2020) Land use history drives differences in functional composition and losses in functional diversity and stability of Neotropical urban forests. Urban For Urban Greening 49:. https://doi.org/10.1016/j.ufug.2020.126608

Quartucci F, Gocke M, Denich M, et al (2023) Deep soil carbon loss offsets rapid aboveground carbon accumulation after reforestation. For Ecol Manage 548:. https://doi.org/10.1016/j.foreco.2023.121403

Robinson SJB, Berg E van den, Meirelles GS, Ostle N (2015) Factors influencing early secondary succession and ecosystem carbon stocks in Brazilian Atlantic Forest. Biodiversity Conserv 24:2273–2291. https://doi.org/10.1007/s10531-015-0982-9

Rodrigues AC, Villa PM, Ferreira-Júnior WG, et al (2021) Effects of topographic variability and forest attributes on fine-scale soil fertility in late-secondary succession of Atlantic Forest. Ecol Processes 10:. https://doi.org/10.1186/s13717-021-00333-1

Rolim SG, Sambuichi RHR, Schroth G, et al (2017) Recovery of Forest and Phylogenetic Structure in Abandoned Cocoa Agroforestry in the Atlantic Forest of Brazil. Environ Manage 59:410–418. https://doi.org/10.1007/s00267-016-0800-5

Rosa MR, Brancalion PHS, Crouzeilles R, et al (2021) Hidden destruction of older forests threatens Brazil’s Atlantic Forest and challenges restoration programs. Sci Adv 7:. https://doi.org/10.1126/sciadv.abc4547

Saad SI, Silva JM da, Ponette-González AG, et al (2021) Modeling the on-site and off-site benefits of Atlantic forest conservation in a Brazilian watershed. Ecosyst Serv 48:. https://doi.org/10.1016/j.ecoser.2021.101260

Sales GPDS, Guedes-Bruni RR (2023) New sources of biological data supporting environmental history of a tropical forest of south-eastern brazil. Hist Ambient Latinoam Caribena 13:281–308. https://doi.org/10.32991/2237-2717.2023V13I2.P281-308

Sansevero JBB, Prieto PV, Sánchez-Tapia A, et al (2017) Past land-use and ecological resilience in a lowland Brazilian Atlantic Forest: implications for passive restoration. New For 48:573–586. https://doi.org/10.1007/s11056-017-9586-4

Sant’Anna CLB, Ribeiro DB, Garcia LC, Freitas AVL (2014) Fruit-feeding butterfly communities are influenced by restoration age in tropical forests. Restor Ecol 22:480–485. https://doi.org/10.1111/rec.12091

Santos AR, Barbosa MAGA, Bolleli T, et al (2023) Assessment of Water Ecosystem Integrity (WEI) in a Transitional Brazilian Cerrado–Atlantic Forest Interface. Water 15:. https://doi.org/10.3390/w15040775

Santos FAM dos, Coelho-Junior MG, Cardoso JC, et al (2020) Program outcomes of payments for watershed services in Brazilian Atlantic forest: How to evaluate to improve decision-making and the socio-environmental benefits. Water 12:. https://doi.org/10.3390/w12092441

Schweizer D, Chazdon RL (2021) Case #3: Bringing the Atlantic Forest back to life in the Rio Doce Watershed, Minas Gerais, Brazil. Restor; Forestation International

Shennan-Farpón Y, Mills M, Souza A, Homewood K (2022) The role of agroforestry in restoring Brazil’s Atlantic Forest: Opportunities and challenges for smallholder farmers. People Nat 4:462–480. https://doi.org/10.1002/pan3.10297

Shennan-Farpón Y, Soterroni AC, Scarabello M, Visconti P (2024) Using policy scenarios to assess challenges and opportunities for reaching restoration targets in Brazil’s Atlantic Forest. EnvironResLett 19:. https://doi.org/10.1088/1748-9326/ad5ab2

Shimamoto CY, Botosso PC, Marques MCM (2014) How much carbon is sequestered during the restoration of tropical forests? Estimates from tree species in the Brazilian Atlantic forest. For Ecol Manage 329:1–9. https://doi.org/10.1016/j.foreco.2014.06.002

Souza SEXF de, Vidal E, Chagas GDF, et al (2016) Ecological outcomes and livelihood benefits of community-managed agroforests and second growth forests in Southeast Brazil. Biotropica 48:868–881. https://doi.org/10.1111/btp.12388

Stoms DM, Chomitz KM, Davis FW (2004) TAMARIN: A landscape framework for evaluating economic incentives for rainforest restoration. Landsc Urban Plann 68:95–108. https://doi.org/10.1016/S0169-2046(03)00169-5

Strassburg BBN, Barros FSM, Crouzeilles R, et al (2016) The role of natural regeneration to ecosystem services provision and habitat availability: a case study in the Brazilian Atlantic Forest. Biotropica 48:890–899. https://doi.org/10.1111/btp.12393

Strassburg BBN, Beyer HL, Crouzeilles R, et al (2019) Strategic approaches to restoring ecosystems can triple conservation gains and halve costs. Nat Ecol Evol 3:62–70. https://doi.org/10.1038/s41559-018-0743-8

Torres FN de, Richter R, Fidalgo ECC (2019) Multicriteria Site Prioritization for Land Rehabilitation in the Guapi-Macacu Watershed, Rio de Janeiro. In: Nehren U, Schlϋter S, Raedig C, et al. (eds) Strategies and Tools for a Sustainable Rural Rio de Janeiro. Springer International Publishing, Cham, pp 405–421

Valente FD de A, Castro MF de, Lustosa Filho JF, et al (2023) Native multispecies and fast-growing forest root biomass increase C and N stocks in a reclaimed bauxite mining area. Environ Monit Assess 195:. https://doi.org/10.1007/s10661-022-10720-6

Valente RA, Mello K de, Metedieri JF, Américo C (2021) A multicriteria evaluation approach to set forest restoration priorities based on water ecosystem services. J Environ Manage 285:. https://doi.org/10.1016/j.jenvman.2021.112049

Vettorazzi CA, Valente RA (2016) Priority areas for forest restoration aiming at the conservation of water resources. Ecological Engineering 94:255–267. https://doi.org/10.1016/j.ecoleng.2016.05.069

Viani RAG, Bracale H, Taffarello D (2019) Lessons learned from the water producer project in the Atlantic Forest, Brazil. Forests 10:. https://doi.org/10.3390/f10111031

Viani RAG, Braga DPP, Ribeiro MC, et al (2018) Synergism Between Payments for Water-Related Ecosystem Services, Ecological Restoration, and Landscape Connectivity Within the Atlantic Forest Hotspot. Trop Conserv Sci 11:. https://doi.org/10.1177/1940082918790222

Vibrans AC, Oliveira LZ, Gasper AL de, et al (2022) Unprecedented large-area turnover estimates for the subtropical Brazilian Atlantic Forest based on systematically-gathered data. For Ecol Manage 505:. https://doi.org/10.1016/j.foreco.2021.119902

Zambiazi DC, Fantini AC, Piotto D, et al (2021) Timber stock recovery in a chronosequence of secondary forests in Southern Brazil: Adding value to restored landscapes. For Ecol Manage 495:. https://doi.org/10.1016/j.foreco.2021.119352

Zwiener VP, Padial AA, Marques MCM, et al (2017) Planning for conservation and restoration under climate and land use change in the Brazilian Atlantic Forest. Diversity Distrib 23:955–966. https://doi.org/10.1111/ddi.12588
